# Differentiation of Tumorigenic C6 Glioma Cells Induced by Enhanced IL-6 Signaling

**DOI:** 10.3390/medicina56110625

**Published:** 2020-11-19

**Authors:** Inn-Ray Chu, Rong-Long Pan, Chung-Shi Yang

**Affiliations:** 1 Department of Life Science and Institute of Bioinformatics and Structural Biology, College of Life Science, National Tsing Hua University, Hsin Chu 30013, Taiwan; yingrui328@gapp.nthu.edu.tw (I.-R.C.); rlpan@life.nthu.edu.tw (R.-L.P.); 2 Institute of Biomedical Engineering and Nanomedicine, National Health Research Institutes, Zhunan, Miaoli 35053, Taiwan

**Keywords:** cancer stem cell, differentiation therapy, interleukin-6, glial fibrillary acidic protein, soluble interleukin-6 receptor, tumor necrosis factor-α

## Abstract

*Background and objectives*: Cancer stem cells (CSCs) are obstacles to cancer therapy due to their therapeutic resistance, ability to initiate neoplasia, and roles in tumor relapse and metastasis. Efforts have been made to cure CSCs, such as the use of differentiation therapy, which induces cancer stem-like cells to undergo differentiation and decrease their tumorigenicity. Interleukin 6 (IL-6) upregulates the expression of glial fibrillary acidic protein (GFAP) in C6 glioma cells, indicating that it is able to induce the differentiation of these cells. The C6 glioma cell line forms a high percentage of cancer stem-like cells, leading us to speculate whether IL-6 signaling could modulate the differentiation of tumorigenic C6 glioma cells. However, we observed that IL-6 alone could not efficiently induce the differentiation of these cells. Therefore, different IL-6 signaling elicitors, including IL-6 alone, a combination of IL-6 and soluble IL-6 receptor (IL-6/sIL-6R), and tumor necrosis factor-α (TNF-α) plus IL-6/sIL-6R (TNF-α/IL-6/sIL-6R), were evaluated for their potential use in differentiation therapy. *Materials and Methods*: The potential of IL-6 signaling elicitors in differentiation therapy were examined by assessing changes in biomarker levels, the rate of cell proliferation, and tumorigenicity, respectively. *Results*: Enhanced IL-6 signaling could effectively induce C6 glioma cell differentiation, as determined by observed variations in the expression of differentiation, cell cycle, and stem cell biomarkers. Additionally, the total cell population and the tumorigenicity of glioma cells were all considerably reduced after TNF-α/IL-6/sIL-6R treatment. *Conclusions*: Our findings provide evidence that enhanced IL-6 signaling can efficiently promote tumorigenic C6 glioma cells to undergo differentiation.

## 1. Introduction

Cancer is the second cause of death in the world, resulting in an estimated 18.1 million new cases and 9.6 million deaths in 2018 [1]. Cancer cells arisen from our own cells have several distinct features in histology, such as a larger nucleus and abnormal cytoplasm, and physiology, such as escape of cell programming death and metastasis [2]. According to the hierarchical model, cancer stem cells (CSCs) represent a subpopulation of tumor cells and are similar to normal stem cells. CSCs can self-renew and differentiate into heterogeneous cell lineages [3,4], playing important roles in maintaining the malignancy of the tumor and in tumor initiation, development, and recurrence [5,6,7]. Thus, the existence of CSCs is an important cause of the failure of regular cancer therapy, including chemo and radiation therapies [8,9].

In recent years, some therapies have been developed against CSCs [10] that are classified into two types depending on whether they exert direct or indirect therapeutic effects on CSCs. The direct strategies include the elimination of CSCs by targeting their specific markers, interference with drug resistance mechanisms, and differentiation therapy. Indirect strategies include the disruption of immune evasion, interruption of the interplay among CSCs and their microenvironments, and anti-angiogenic therapy. Differentiation therapy is a potential method for curing CSCs by which CSCs are induced to differentiate to alleviate their tumorigenicity [10]. This strategy makes CSCs more sensitive to chemo and radiation therapies [10]. Thus far, many successful studies have demonstrated the effects of differentiation therapy on CSCs [11,12,13,14]. For instance, bone morphogenetic protein4 (BMP4) induces Smad signaling in human glioblastoma cells, which upregulates the expression of neural differentiation markers, inhibits cellular proliferation, and reduces the size of the tumorigenic CD133^+^ population [11]. This evidence indicates that BMP4 acts as an inhibitory regulator of brain CSCs.

Interleukin-6 (IL-6) has been suggested to induce the astrocytic differentiation of C6 glioma cells [15]. The role of IL-6 signaling was confirmed in the cyclic adenosine monophosphate (cAMP) induced differentiation of C6 glioma cells [15]. When IL-6 signaling was blocked by an IL-6-specific antibody, this differentiation process could no longer be observed. Moreover, the expression of the astrocyte-specific marker glial fibrillary acidic protein (GFAP) was also diminished after this treatment. Later, studies showed that phosphorylated signal transducer and activator of transcription 3 (p-STAT3) in the IL-6 signaling pathway could bind to the GFAP promoter as a transcription factor to initiate its transcription [16]. In addition, the influence of IL-6 signaling on the differentiation of C6 glioma cells has been mentioned elsewhere [17,18].

IL-6 is a pleiotropic cytokine that was initially discovered as a factor named B cell stimulatory factor-2 (BSF-2), produced by T cells to stimulate immunoglobulin production by B cells [19]. IL-6 regulates cell growth, proliferation, survival, and differentiation, and its roles in a variety of other processes have also been explored, including immune responses, neurotrophic effects, neuroprotective effects, inflammation and anti-inflammation reactions, hematopoiesis, and oncogenesis [20,21,22,23,24]. The IL-6 receptor (IL-6R) is composed of two subunits, IL-6Rα and glycoprotein 130 (gp130) [25]. IL-6Rα is a membrane-bound protein that specifically binds IL-6, while gp130 acts as a signal transducer that is used by other cytokines in the IL-6 family [25]. Upon IL-6 binding to IL-6Rα, the resulting complex associates with gp130 to initiate intrinsic cellular signaling pathways, such as the Janus kinase (JAK)/STAT, Ras/mitogen-activated protein kinase (MAPK), and phosphatidylinositol-3-kinase (PI3K)/AKT/nuclear factor kappaB (NF-κB) pathways [25].

Additionally, clonal and population analyses have shown that most C6 glioma cells, including CD133^−^ and non-side population cells, possess self-renewal, clonogenic, and tumorigenic capabilities [26]. C6 glioma cells have been cultured in vitro for decades and retain stable tumorigenicity. Thus, if a C6 cell population contains a low percentage of cancer stem-like cells, this subpopulation will ultimately disappear from the cell mass [26]. Based on these findings, we speculated that IL-6 signaling likely induces the differentiation of tumorigenic C6 glioma cells to reduce their tumorigenicity. Nevertheless, after pretreating C6 glioma cells [27] with recombinant IL-6 protein, no obvious differentiation was observed. Subsequently, we examined whether increased IL-6 signaling could effectively induce the differentiation of this cell line as a possible approach for cancer therapy.

A synergistic effect in upregulating the production of IL-6 has been reported for tumor necrosis factor-α (TNF-α), IL-6, and sIL-6R [28]. RT-PCR results showed that the level of *Il-6* mRNA in U373-MG glioma cells was elevated approximately 1-, 2-, and 10-fold by IL-6, a combination of IL-6 and soluble IL-6 receptor (IL-6/sIL-6R), and TNF-α plus IL-6 and soluble IL-6 receptor (TNF-α/IL-6/sIL-6R), respectively [28]. Our results indicated that TNF-α/IL-6/sIL-6R can efficiently upregulate the expression of differentiation markers and downregulate that of cell cycle and stem cell markers. The use of other stimulators of IL-6 signaling, including IL-6 and IL-6/sIL-6R, elicited lower levels of glioma cell differentiation. Moreover, TNF-α/IL-6/sIL-6R also decreased the total cell population and the tumorigenicity of glioma cells. We observed that a higher dose of IL-6 signaling could effectively induce the differentiation of C6 glioma cells.

## 2. Materials and Methods

### 2.1. Cell Cultures

The rat C6 glioma cell line was obtained from the National Health Research Institute Cell Bank (Zhunan, Taiwan). The tumorigenicity of this C6 rat glioma cell line has been previously evaluated [27]. The cells were cultured in DMEM/F12 supplemented with 10% (*w*/*v*) FBS, 1% (*w*/*v*) penicillin, and streptomycin (Invitrogen, Carlsbad, CA, USA) in a humidified atmosphere with 5% CO_2_ at 37 °C.

### 2.2. RNA Extraction and Reverse Transcription-Polymerase Chain Reaction (RT-PCR)

C6 glioma cells were plated into six-well plates at a density of 1.7 × 10^5^ cells/well. After being treated with IL-6 signaling elicitors, including IL-6, IL-6/sIL-6R, and TNF-α/IL-6/sIL-6R (TNF-α: 20 ng/mL, IL-6: 5 ng/mL, and sIL-6R: 100 ng/mL; all from PeproTech, Rocky Hill, NJ, USA), for two days, the total RNA of the treated cultures was isolated using the Total RNA Extraction Miniprep System (Viogene, New Taipei City, Taiwan) according to the manufacturer’s instructions and quantified by measuring the absorbance at 260 nm (Nanodrop 1000; Thermo Fisher Scientific, Wilmington, DE, USA). Five micrograms of total RNA from different experimental groups were then reverse transcribed using the SuperScript III First-Strand Synthesis System (Invitrogen) following the manufacturer’s instructions. One microliter (250 ng) of newly synthesized complementary DNA (cDNA) from each group was amplified in a 50 μL reaction mixture comprising 5 μL of 10× Taq buffer (Protech, Taipei, Taiwan), 1 μL of 10 mM dNTPs (Protech), 1 μL of 100 μM forward primer (MDBio, Taipei, Taiwan), 1 μL of 100 μM reverse primer (MDBio), and 0.5 μL of Pro Taq DNA polymerase (Protech) using a T100 thermal cycler (Bio-Rad, Hercules, CA, USA) (Table 1 and Table 2).

### 2.3. Western Blot Analysis

After being treated with IL-6 signaling elicitors, including IL-6, IL-6/sIL-6R, and TNF-α/IL-6/sIL-6R (TNF-α: 20 ng/mL, IL-6: 5 ng/mL, and sIL-6R: 100 ng/mL), for two days, C6 glioma cells were lysed using CelLytic M (Sigma-Aldrich, St. Louis, MO, USA) and centrifuged at 15,000× *g* for 15 min at 4 °C. Protein concentrations were measured by the Bradford method (Bio-Rad) according to the manufacturer’s instructions. Afterward, 20 μg of proteins from different experimental groups were separated by 10% SDS-PAGE and transferred to polyvinylidene fluoride (PVDF) membranes (Bio-Rad), followed by blocking with 5% (*w*/*v*) non-fat milk for 1 h at room temperature. The membranes were then incubated with specific primary antibodies against STAT3 (catalog no. 610189, 1:2000 dilution; BD Biosciences, San Jose, CA, USA), p-STAT3 (catalog no. sc-8059, 1:50 dilution; Santa Cruz Biotechnology, Dallas, TX, USA), epithelial cadherin (E-cadherin; catalog no. sc-7870, 1:500 dilution; Santa Cruz Biotechnology), connexin-43 (catalog no. #3512, 1:2000 dilution; Cell Signaling Technology, Danvers, MA, USA), GFAP (catalog no. 04-1062, 1:100 dilution; EMD Millipore, Billerica, MA, USA), telomerase reverse transcriptase (TERT; catalog no. sc-7212, 1:100 dilution; Santa Cruz Biotechnology), proliferating cell nuclear antigen (PCNA; catalog no. sc-25280, 1:2000 dilution; Santa Cruz Biotechnology), nestin (catalog no. MAB353, 1:600 dilution; Merck Millipore, Darmstadt, Germany), musashi-1 (Msi-1; catalog no. sc-135721, 1:100 dilution; Santa Cruz Biotechnology), and β-actin (catalog no. MAB1501, 1:3000 dilution; Merck Millipore) overnight at 4 °C. After washing with TBS with Tween, the membranes were treated with appropriate horseradish peroxidase labeled secondary antibodies (anti-mouse IgG: catalog no. sc-2005, 1:10,000 dilution and anti-rabbit IgG: catalog no. sc-2357, 1:10,000 dilution; Santa Cruz Biotechnology), then the Western blots were incubated with ECL detection reagents (Thermo Fisher Scientific). Finally, the bands were visualized using a UVP BioSpectrum image system (UVP, Upland, CA, USA).

### 2.4. Dye-Coupling Assay

A dye-coupling assay was performed to evaluate the gap junction communication between C6 glioma cells. After treatment with IL-6 signaling elicitors, including IL-6 and TNF-α/IL-6/sIL-6R (TNF-α: 20 ng/mL, IL-6: 5 ng/mL, and sIL-6R: 100 ng/mL), for two days, donor cells (TNF-α/IL-6/IL-6R-treated and untreated cells) were incubated for 20 min with a dual label dye solution containing 0.3 M of isotonic PBS-glucose, 10 µM of DiI (Invitrogen), and 5 µM of calcein-AM (Invitrogen). Afterward, these cells were rinsed three times with PBS-glucose, trypsinized, suspended in the growth medium, and seeded onto confluent recipient cells (TNF-α/IL-6/sIL-6R-treated cells) at a ratio of 1 to 500. After incubating for 4 h, gap junction communication was assessed by the transfer of calcein from donor to recipient cells through fluorescence microscopy.

### 2.5. Cell Proliferation Assay

Cells were seeded into 96-well plates at a density of 200 cells/well in 100 µL of culture medium containing various IL-6 signaling elicitors, including IL-6, IL-6/sIL-6R, and TNF-α/IL-6/sIL-6R (TNF-α: 20 ng/mL, IL-6: 5 ng/mL, and sIL-6R: 100 ng/mL), for five days. The total cell population of each group was measured daily by a WST-1 assay (Roche, Indianapolis, IN, USA). The WST-1 reagent (10 µL/well) was added into culture medium and incubated for 4 h at 37 °C and 5% CO_2_. Afterward, the absorbance of the samples against a background control as a blank was measured at 450 nm.

### 2.6. Apoptosis and Necrosis Detection

After being treated with IL-6 signaling elicitors, including IL-6, IL-6/sIL-6R, and TNF-α/IL-6/sIL-6R (TNF-α: 20 ng/mL, IL-6: 5 ng/mL, and sIL-6R: 100 ng/mL), for five days, TNF-α/IL-6/sIL-6R-treated cells were collected, washed, and resuspended in 1× Binding Buffer (BD Biosciences) at a concentration of 1 × 10^6^ cells/mL. Subsequently, cells were stained with annexin V (catalog no. 51-65874X; BD Biosciences) and propidium iodide (PI) (catalog no. 51-66211E; BD Biosciences) according to the manufacturer’s protocols. The stained cells were observed using a FACSCalibur flow cytometry system (BD Biosciences) to detect apoptosis and necrosis events.

### 2.7. Soft Agar Assay

After IL-6 signaling elicitor treatment, including IL-6 and TNF-α/IL-6/sIL-6R (TNF-α: 20 ng/mL, IL-6: 5 ng/mL, and sIL-6R: 100 ng/mL), for two days, cells were filtered through 40-μm Falcon strainers (BD Biosciences) and seeded into the upper layer of 0.3% (*w*/*v*) noble agar (Sigma-Aldrich) in six-well plates at a density of 2500 cells/well to evaluate their tumorigenicity [29]. Seven days later, the diameters of the tumor colonies (25 random 200× fields) were measured using DP2-BSW imaging software (Olympus, Tokyo, Japan). Because not all of the colonies were spherical, the maximal diameter of each colony was determined. Cells were considered tumorigenic based on the number of colonies with a diameter of >50 μm. Subsequently, the cells were photographed after another incubation period of seven days.

### 2.8. Sphere Formation Assay

After being treated with IL-6 signaling elicitors, including IL-6 and TNF-α/IL-6/sIL-6R (TNF-α: 20 ng/mL, IL-6: 5 ng/mL, and sIL-6R: 100 ng/mL), for two days, cells were trypsinized, suspended in a serum-free medium, and filtered through 40-μm Falcon strainers (BD Biosciences). Subsequently, a single-cell suspension was prepared (10 cells/mL), seeded in 96-well plates (100 μL per well), and cultured in a serum-free medium supplemented with 20 ng/mL of basic fibroblast growth factor (bFGF; Sigma-Aldrich), 20 ng/mL of epidermal growth factor (EGF; Sigma-Aldrich), and 20 µL/mL of B27 supplement (Invitrogen). Afterward, wells containing only one cell were labeled sequentially from 1 to 40 and observed daily, while wells without cells or containing more than one cell were not included in the analysis. Six days later, the diameters of the floating spheres were determined using DP2-BSW imaging software (Olympus). The cells were considered tumorigenic if spheres with a diameter of >40 μm were observed.

### 2.9. Statistical Analysis

Statistical analysis was performed using IBM SPSS Statistics version 26 (IBM Corporation, Armonk, NY, USA). All data are presented as the mean ± SD of at least three independent experiments or are representative of experiments repeated at least three times. One-way ANOVA followed by Tukey’s post hoc test was employed to evaluate differences among distinct experimental groups in the cell proliferation assay. In addition, the statistical significance of results from the flow cytometric analysis, soft agar assay, and sphere formation assay were analyzed by the paired Student’s *t*-test; *p* < 0.05 was considered to indicate a statistically significant difference.

## 3. Results

### 3.1. The Expression of Il-6 Is Efficiently Triggered by TNF-α/IL-6/sIL-6R

**Figure 1:**
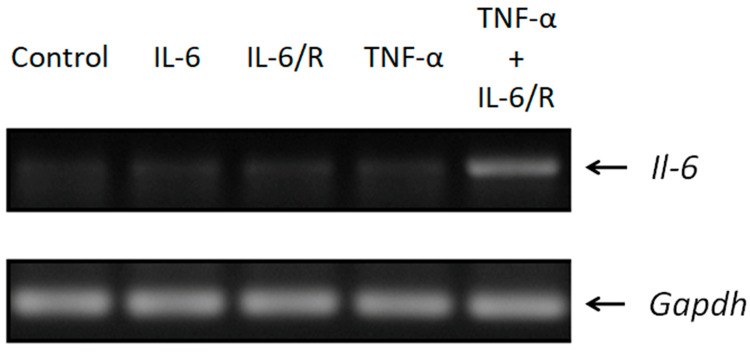
Upregulation of *Il-6* in C6 glioma cells. Glioma cells were treated with IL-6, IL-6/sIL-6R, and TNF-α/IL-6/sIL-6R and examined for *Il-6* and *Gapdh* gene expression via RT-PCR, respectively. IL-6/R represents IL-6/sIL-6R.

We examined the expression of the *Il-6* gene in a tumorigenic C6 glioma cell line treated with IL-6, IL-6/sIL-6R, and TNF-α/IL-6/sIL-6R. Two days later, the mRNA levels of *Il-6* and glyceraldehyde-3-phosphate dehydrogenase (*Gapdh*) were assessed by RT-PCR (Table 1). The results revealed that TNF-α/IL-6/sIL-6R could efficiently upregulate the expression of *Il-6* more than IL-6/sIL-6R or IL-6 alone in glioma cells (Figure 1).

### 3.2. Differentiation of C6 Glioma Cells Is Induced by TNF-α/IL-6/sIL-6R as Evidenced by Changes in Biomarker Levels

**Figure 2:**
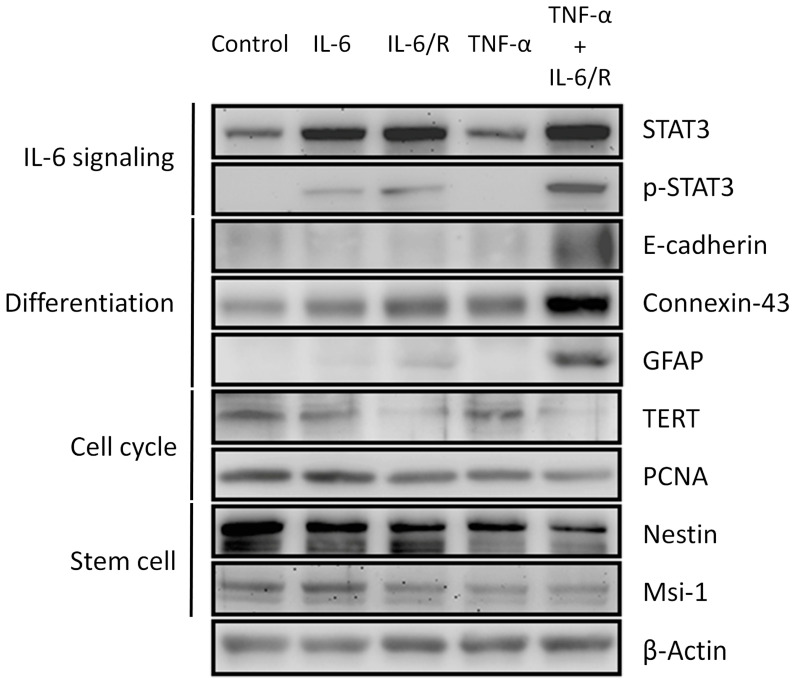
The differentiation state of C6 glioma cells determined according to biomarkers. Western blots of glioma cells were carried out after cytokine treatment. Biomarkers for IL-6 signaling (STAT3 and p-STAT3), differentiation (E-cadherin, connexin-43, and GFAP), cell cycle (TERT and PCNA), and stem cells (nestin and Msi-1) were monitored. IL-6/R represents IL-6/sIL-6R.

We treated glioma cells with various cytokine complexes for two days and used Western blotting to assess the expression of biomarkers. The evaluated biomarkers can be classified into four groups, including those for IL-6 signaling (STAT3 and p-STAT3), differentiation (E-cadherin, connexin-43, and GFAP), cell cycle (TERT and PCNA), and stem cell (nestin and Msi-1) [30,31,32,33,34,35,36,37,38,39]. The results showed that TNF-α/IL-6/sIL-6R upregulated the levels of markers of IL-6 signaling and differentiation, but downregulated those of cell cycle and stem cell markers (Figure 2). IL-6/sIL-6R and IL-6 respectively elicited moderate and minor effects on these markers. Therefore, we demonstrated that TNF-α/IL-6/sIL-6R can differentiate C6 glioma cells more efficiently than IL-6/sIL-6R and IL-6 alone.

**Figure 3:**
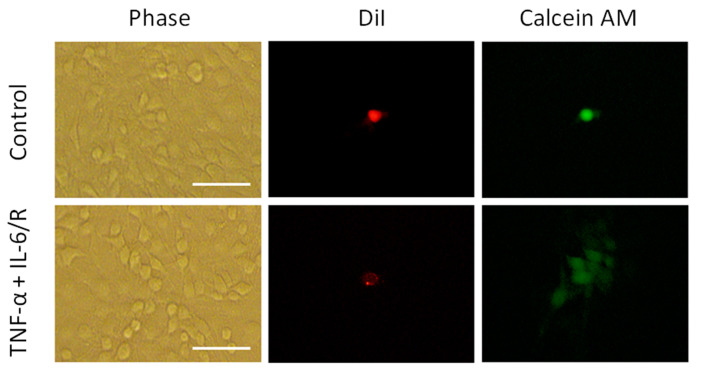
The upregulation of gap junction in C6 glioma cells. Demonstration of the formation of gap junctions between the calcein donor and recipient cells through a dye-coupling assay. Donor cells labeled with DiI and calcein-AM were seeded onto confluent recipient cells, and the transfer of green fluorescent calcein from donor to neighboring cells was evaluated. Scale bar, 80 μm. IL-6/R represents IL-6/sIL-6R.

Subsequently, we further confirmed the expression of differentiation markers in TNF-α/IL-6/sIL-6R treated glioma cells. We applied a dye-coupling assay to examine the communication at gap junctions. TNF-α/IL-6/sIL-6R treated and untreated cells were labeled with DiI and calcein-AM as donor cells. After incubation with recipient cells (TNF-α/IL-6/sIL-6R treated cells), the establishment of gap junctions between these two cell types was evaluated by assessing the transfer of green fluorescent calcein from donor to neighboring cells (Figure 3). The results showed that, compared to control cells, TNF-α/IL-6/sIL-6R treated glioma cells formed more gap junctions with nearby cells.

### 3.3. TNF-α/IL-6/sIL-6R Decreases the Proliferation Rate of C6 Glioma Cells

**Figure 4:**
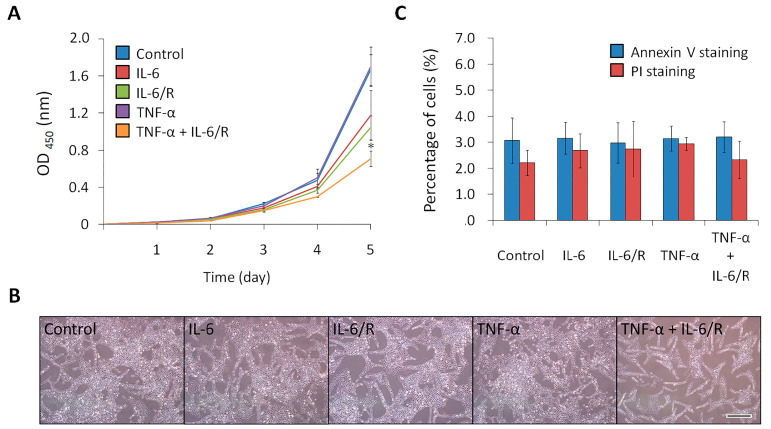
The proliferation rate of C6 glioma cells reduced by TNF-α/IL-6/sIL-6R. (**A**) The total cell population of each cytokine-treated group. Glioma cells were treated with cytokines in various groups. The cell population was measured daily for five days using a WST-1 proliferation assay, and the absorbance was measured at 450 nm. (**B**) Images taken on day three. Scale bar, 500 μm. (**C**) Detection of apoptosis and necrosis. Glioma cells were stained with annexin V and PI after cytokine treatment for five days and detected by flow cytometric analysis. The statistical results are the mean of three experiments ± SD, and the asterisk indicates significant differences (*p* < 0.05) between the TNF-α/IL-6/sIL-6R treated group and control group. IL-6/R represents IL-6/sIL-6R.

Differentiated cells typically exhibit a lower cell proliferation rate compared to tumorigenic cells. After C6 glioma cells were treated with IL-6 signaling elicitors, the total cell populations were measured daily by the WST-1 assay for five days, with images taken on day three. The results showed that the total cell population of the TNF-α/IL-6/sIL-6R treated group was less than that of the other groups and approximately 42.4 ± 5.1% (mean ± SD, *n* = 3) of the control (Figure 4A,B). In addition, glioma cells labeled with annexin V and PI on day five did not exhibit obvious apoptosis or necrosis (Figure 4C). As a result, differentiation was shown to be a decisive factor causing variation in the total cell number among the different groups. These results showed that C6 glioma cells could be efficiently differentiated by TNF-α/IL-6/sIL-6R to decrease their proliferation rate.

### 3.4. TNF-α/IL-6/sIL-6R Reduces the Tumorigenicity of C6 Glioma Cells

**Figure 5:**
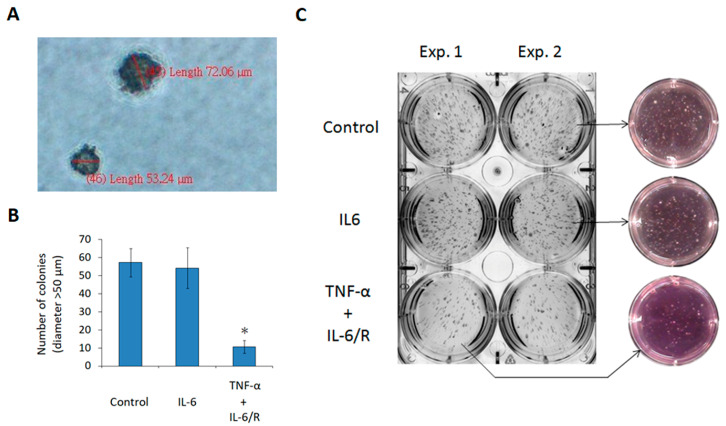
Downregulation of the tumorigenicity in C6 glioma cells evidenced by the soft agar assay. (**A**) The diameters of colonies. Cytokine-treated cells were seeded in 0.3% (*w*/*v*) noble agar. After one week of incubation, the diameters of the colonies were measured using DP2-BSW imaging software. (**B**) Number of colonies with a diameter of >50 μm. (**C**) Images of colonies taken after a two-week incubation. The color of phenol red was inversely proportional to the total cell mass in the wells. The statistical results are the mean of three experiments ± SD, and the asterisk indicates significant differences (*p* < 0.05) between the TNF-α/IL-6/sIL-6R treated group and control group. IL-6/R represents IL-6/sIL-6R.

Finally, the tumorigenicity of glioma cells was evaluated after treatment with IL-6 signaling inducers. The soft agar assay is used to assess cellular anchorage-independent growth and tumorigenicity [29]. Glioma cells were processed with cytokines and seeded in the gel with a low percentage agar for one week of culturing. The diameters of the colonies were measured (Figure 5A), and the number of colonies with a diameter >50 μm was determined. The results revealed that TNF-α/IL-6/sIL-6R treated cells formed fewer colonies (10.7 ± 3.5; mean ± SD, *n* = 3) than control cells (57.3 ± 7.8; mean ± SD, *n* = 3) (Figure 5B). Subsequently, after incubating for another week, there were fewer colonies observed in the TNF-α/IL-6/sIL-6R treated group than in the other groups. The color of the pH indicator phenol red was inversely proportional to the total cell mass in the well (Figure 5C).

**Figure 6:**
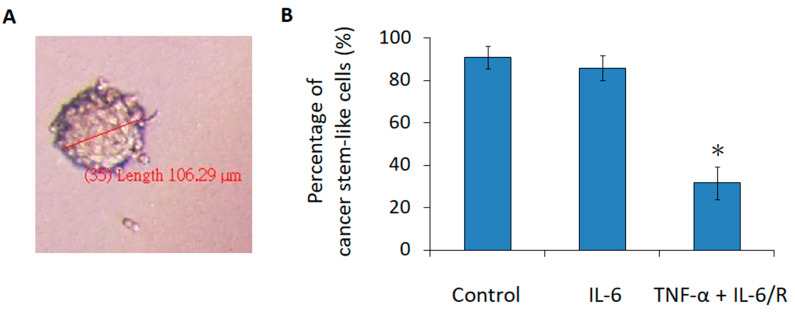
Downregulation of the tumorigenicity in C6 glioma cells evidenced by the sphere formation assay. (**A**) The diameters of the spheres measured using DP2-BSW imaging software. (**B**) Percentage of cancer stem-like cells with a diameter of >40 µm. The statistical results are the mean of three experiments ± SD, and the asterisk indicates significant differences (*p* < 0.05) between the TNF-α/IL-6/sIL-6R treated group and control group. The total numbers for each group were 40 spheres. IL-6/R represents IL-6/sIL-6R.

Additionally, the sphere formation assay was used to measure the tumorigenicity of cancer cells in vitro [40]. In this assay, if a single cancer cell can form a floating sphere in a neural stem cell (NSC) culture medium, then this cell is considered to be tumorigenic. After treatment with IL-6 signaling elicitors, glioma cells were seeded at one cell per well and allowed to grow for 6 days. The diameters of the spheres were measured (Figure 6A), and the number of spheres with a diameter of >40 μm was determined; TNF-α/IL-6/sIL-6R treated cells formed fewer spheres (31.7 ± 7.6%; mean ± SD, *n* = 3) than control cells (90.8 ± 5.2%; mean ± SD, *n* = 3) (Figure 6B). Furthermore, the diameters of most spheres (68.3 ± 7.6%; mean ± SD, *n* = 3) in the TNF-α/IL-6/sIL-6R treated group were 0–40 μm, whereas those (36.7 ± 7.6%; mean ± SD, *n* = 3) in the control group were 80–120 μm (Figure S1). Taking these two assays into consideration, it was concluded that TNF-α/IL-6/sIL-6R treatment decreased the tumorigenicity of the C6 glioma cell line.

## 4. Discussion

Herein, we confirmed that increased IL-6 signaling could induce tumorigenic C6 glioma cells to undergo differentiation by assessing changes in biomarker levels, the rate of cell proliferation, and tumorigenicity. In TNF-α/IL-6/sIL-6R treated glioma cells, STAT3 can be phosphorylated (p-STAT3) as a transcription factor to participate in IL-6 signaling. In addition, the levels of E-cadherin, connexin-43, and GFAP, which are abundantly expressed in astrocytes and at a low level in glioma cells, were upregulated; p-STAT3 has been found to initiate the promoter of GFAP [16]. Moreover, the levels of biomarkers for the cell cycle (TERT and PCNA) and NSC (Nestin and Msi-1) were consistently downregulated, and agreed with the cell proliferation rate results and tumorigenicity evaluations.

The TNF-α/IL-6/sIL-6R complex could more efficiently induce the expression of *Il-6* than IL-6/sIL-6R or IL-6 alone in a tumorigenic C6 glioma cell line. This complex gave rise to increased IL-6 signaling, which might result from much stronger IL-6 autocrine signaling. The pro-inflammatory cytokine TNF-α has been shown to induce IL-6 expression via the phosphorylation of NF-κB, p38 MAPK, and stress-activated protein kinase (SAPK)/c-Jun N-terminal kinase (JNK) [41]. As a result, secreted IL-6 could bind to the extracellular sIL-6R and then associate with membrane-bound gp130 to initiate intrinsic IL-6 signaling in glioma cells. Furthermore, exogenous sIL-6R plays an important role in inducing increased IL-6 signaling due to the limited amount of IL-6Rα. We applied 100 ng/mL of IL-6-treated tumorigenic C6 glioma cells to conduct the sphere formation assay, but the results did not show significant differences compared to the cytokine-untreated group (unpublished data).

Further in vivo studies are currently underway in our research laboratory to elevate the clinical application of this strategy. To this end, three conditions are taken into account before setting up experiments. First, two commonly defined CSC models, stochastic and hierarchical, may be able to describe CSCs due to the bi-directional interconvertibility between CSCs and non-CSCs through the epithelial to mesenchymal transition (EMT) and the reciprocal mesenchymal to epithelial transition (MET) [42,43]. Second, it is not easy to maintain glioma cells in a TNF-α/IL-6/sIL-6R contained micro-environment in the rat brain for differentiation therapy. Furthermore, TNF-α and IL-6 are multi-functional cytokines associated with many signaling pathways. As a result, tumor localized treatment is a better strategy to induce the differentiation of tumorigenic C6 glioma cells in vivo. Lastly, we have elaborated that TNF-α/IL-6/sIL-6R could downregulate the proliferation rate and the tumorigenicity of glioma cells, but not eliminate them. The results of sphere formation assays showed that 31.7 ± 7.6% (mean ± SD, *n* = 3) of glioma cells still could form >40 um spheres after TNF-α/IL-6/sIL-6R treatment (Figure 6B). To deal with these concerns, it was speculated that NSC-based combination gene therapy may be one of the suitable strategies for further orthotopic animal experiments. NSC can presumably track and infiltrate the glioma tumor by inherent tumor-tropic properties [44]. Therefore, the concentrations of therapeutic proteins produced by NSCs are elevated in the site of malignancy. Furthermore, previous studies have revealed that combination gene therapies exert additive or synergistic effects on brain tumors [45,46].

IL-6 signaling also plays important roles in the neurogenesis and gliogenesis of NSCs [16,47,48,49]. However, some controversial evidence has been published showing the opposite effects of IL-6 in the differentiation of NSCs and brain CSCs [50,51]. This conflict may arise from several possibilities. Similar to other cytokines, IL-6 exerts different effects in various physiological microenvironments depending on the strength of IL-6 signaling, the IL-6 receptor type (membrane-bound or soluble), and the cross-talk among IL-6 and other signaling molecules in the stem cell niche, such as TNF-α and IL-1β [23,52,53,54]. In addition, intrinsic differences exist between established glioma cell lines and primary tumor cells [55,56]. Moreover, it is difficult to precisely define the CSC population in primary malignant tumors. CD133, a surface marker of normal human neural precursors, is used to isolate CSCs from primary brain tumor cells and other cancer cells, such as melanoma, osteosarcoma, hepatoma, colorectal cancer, gastric cancer, and breast cancer cells [40,57]. This methodology is challenged by the fact that not only CD133^+^ but also CD133^-^ brain cancer cells can initiate tumor formation as cancer stem-like cells, and these cells have different molecular profiles and growth characteristics compared to CD133^+^ cells [58]. Consequently, the established therapies based on the unique characteristics of isolated CD133^+^ cells may not lead to a complete cancer cure, potentially resulting in tumor recurrence. Thus, this model should not be the sole criterion to assess the features of CSCs in the brain.

Since the first isolation of cancer stem-like cells from leukemia two decades ago, many studies have investigated this cell population in various tumors, including breast, brain, melanoma, prostate, ovarian, colon, pancreatic, liver, lung, and gastric cancers [59]. CSCs have been hypothesized to be a small fraction of malignant cells, and can self-renew and differentiate into multilineage progenies [60,61]. Recently, it has been suggested that tumors may consist of several sub-clones, each containing their own CSCs with distinct phenotypic or genetic features [62]. Therefore, the objective of future cancer therapies is to cure the CSCs of each sub-clone in malignancies. In this way, some therapies designed to target specific markers of CSCs may no longer be suitable, because they are unable to remove all of the CSCs from the tumor. Nonetheless, differentiation therapy, which involves decreasing the tumorigenicity of CSCs by inducing them to undergo the differentiation process, is still a promising choice for CSC therapy.

## 5. Conclusions

In this study, we provide an alternative method by which tumorigenic C6 glioma cells can be efficiently differentiated by enhanced IL-6 signaling. This strategy would be a potential candidate to cure CSCs in the CNS.

## Figures and Tables

**Table 1:** PCR primers specific for *Il-6* and *Gapdh.*

Target Gene	Forward Primer, 5′ to 3′	Reverse Primer, 5′ to 3′	Tm (°C)
*Il-6*	GTCTCGAGATGAAGTTTCTCTCCGCA	GTGGATCCCTAGTGCCGAGTAGA	50
*Gapdh*	TGCACCACCAACTGCTTA	GGACAGGGATGATGTTC	50

**Table 2:** Steps of PCR amplification.

Step		Temperature (°C)	Time	Number of Cycles
1	Initial denaturation	94	5 min	1
	Denaturation	94	30 s	
2	Annealing	50	30 s	30
	Extension	72	45 s	
3	Final extension	72	10 min	1

## Supplementary Materials

The following are available online at https://www.mdpi.com/1010-660X/56/11/625/s1, Figure S1: The size distribution of spheres.

## References

[1] Bray F., Ferlay J., Soerjomataram I., Siegel R.L., Torre L.A., Jemal A. (2018). Global cancer statistics 2018: GLOBOCAN estimates of incidence and mortality worldwide for 36 cancers in 185 countries. CA Cancer J. Clin..

[2] Baba A.I., Câtoi C. (2007). Chapter 3 Tumor Cell Morphology. Comparative Oncology.

[3] Weissman I.L., Morrison S.J., Clarke M.F., Weissman I.L. (2001). Stem cells, cancer, and cancer stem cells. Nat. Cell Biol..

[4] Polyak K., Hahn W.C. (2006). Roots and stems: Stem cells in cancer. Nat. Med..

[5] Bomken S., Fišer K., Heidenreich O., Vormoor J. (2010). Understanding the cancer stem cell. Br. J. Cancer.

[6] Ghiaur G., Gerber J., Jones R.J. (2012). Concise Review: Cancer Stem Cells and Minimal Residual Disease. Stem Cells.

[7] Papaccio F., Paino F., Regad T., Papaccio G., Desiderio V., Tirino V. (2017). Concise Review: Cancer Cells, Cancer Stem Cells, and Mesenchymal Stem Cells: Influence in Cancer Development. Stem Cells Transl. Med..

[8] Donnenberg V.S., Donnenberg A.D. (2005). Multiple Drug Resistance in Cancer Revisited: The Cancer Stem Cell Hypothesis. J. Clin. Pharmacol..

[9] Rich J.N. (2007). Cancer Stem Cells in Radiation Resistance: Figure. Cancer Res..

[10] Frank N.Y., Schatton T., Frank M.H. (2010). The therapeutic promise of the cancer stem cell concept. J. Clin. Investig..

[11] Piccirillo S.G.M., Reynolds B.A., Zanetti N., Lamorte G., Binda E., Broggi G., Brem H., Olivi A., DiMeco F., Vescovi A.L. (2006). Bone morphogenetic proteins inhibit the tumorigenic potential of human brain tumour-initiating cells. Nat. Cell Biol..

[12] Lotem J., Sachs L.M. (2006). Epigenetics and the plasticity of differentiation in normal and cancer stem cells. Oncogene.

[13] Yu F., Yao H., Zhu P., Zhang X., Pan Q., Gong C., Huang Y., Hu X., Su F., Lieberman J. (2007). let-7 Regulates Self Renewal and Tumorigenicity of Breast Cancer Cells. Cell.

[14] Gupta P.B., Onder T.T., Jiang G., Tao K., Kuperwasser C., Weinberg R.A., Lander E.S. (2009). Identification of Selective Inhibitors of Cancer Stem Cells by High-Throughput Screening. Cell.

[15] Takanaga H., Yoshitake T., Hara S., Yamasaki C., Kunimoto M. (2004). cAMP-induced Astrocytic Differentiation of C6 Glioma Cells Is Mediated by Autocrine Interleukin-6. J. Biol. Chem..

[16] Taga T., Fukuda S. (2005). Role of IL-6 in the Neural Stem Cell Differentiation. Clin. Rev. Allergy Immunol..

[17] Takanaga H., Yoshitake T., Yatabe E., Hara S., Kunimoto M. (2004). beta-Naphthoflavone disturbs astrocytic differentiation of C6 glioma cells by inhibiting autocrine interleukin-6. J. Neurochem..

[18] Shu M., Zhou Y., Zhu W., Wu S., Zheng X., Yan G. (2011). Activation of a pro-survival pathway IL-6/JAK2/STAT3 contributes to glial fibrillary acidic protein induction during the cholera toxin-induced differentiation of C6 malignant glioma cells. Mol. Oncol..

[19] Hirano T., Yasukawa K., Harada H., Taga T., Watanabe S., Matsuda T., Kashiwamura S.-I., Nakajima K., Koyama K., Iwamatsu A. (1986). Complementary DNA for a novel human interleukin (BSF-2) that induces B lymphocytes to produce immunoglobulin. Nat. Cell Biol..

[20] Gruol D.L., Nelson T.E. (1997). Physiological and pathological roles of interleukin-6 in the central nervous system. Mol. Neurobiol..

[21] Kishimoto T. (2010). IL-6: From its discovery to clinical applications. Int. Immunol..

[22] Hama T., Miyamoto M., Tsukui H., Nishio C., Hatanaka H. (1989). Interleukin-6 as a neurotrophic factor for promoting the survival of cultured basal forebrain cholinergic neurons from postnatal rats. Neurosci. Lett..

[23] Scheller J., Chalaris A., Schmidt-Arras D., Rose-John S. (2011). The pro- and anti-inflammatory properties of the cytokine interleukin-6. Biochim. Biophys. Acta Bioenerg..

[24] Hoesel B., Schmid J.A. (2013). The complexity of NF-κB signaling in inflammation and cancer. Mol. Cancer.

[25] Heinrich P.C., Behrmann I., Haan S., Hermanns H.M., Müller-Newen G., Schaper F. (2003). Principles of interleukin (IL)-6-type cytokine signalling and its regulation. Biochem. J..

[26] Zheng X., Shen G., Yang X., Liu W. (2007). Most C6 Cells Are Cancer Stem Cells: Evidence from Clonal and Population Analyses. Cancer Res..

[27] Fang K.-M., Yang C.-S., Lin T.-C., Chan T.-C., Tzeng S.-F. (2014). Induced interleukin-33 expression enhances the tumorigenic activity of rat glioma cells. Neuro-Oncology.

[28] Van Wagoner N.J., Oh J.-W., Repovic P., Benveniste E.N. (1999). Interleukin-6 (IL-6) Production by Astrocytes: Autocrine Regulation by IL-6 and the Soluble IL-6 Receptor. J. Neurosci..

[29] Borowicz S., Van Scoyk M., Avasarala S., Rathinam M.K.K., Tauler J., Bikkavilli R.K., Winn R.A. (2014). The Soft Agar Colony Formation Assay. J. Vis. Exp..

[30] Niehof M., Streetz K., Rakemann T., Bischoff S.C., Manns M.P., Horn F., Trautwein C. (2000). Interleukin-6-induced Tethering of STAT3 to the LAP/C/EBPβ Promoter Suggests a New Mechanism of Transcriptional Regulation by STAT. J. Biol. Chem..

[31] Vleminckx K., Vakaet L., Mareel M., Fiers W., Van Roy F. (1991). Genetic manipulation of E-cadherin expression by epithelial tumor cells reveals an invasion suppressor role. Cell.

[32] Yu S., Xiao H.-L., Jiang X.-F., Wang Q.-L., Li Y., Yang X.-J., Ping Y.-F., Duan J.J., Jiang J.-Y., Ye X.-Z. (2012). Connexin 43 Reverses Malignant Phenotypes of Glioma Stem Cells by Modulating E-Cadherin. Stem Cells.

[33] Huang R.P., Hossain M.Z., Sehgal A., Boynton A.L. (1999). Reduced connexin43 expression in high-grade human brain glioma cells. J. Surg. Oncol..

[34] Soroceanu L., Manning T.J., Sontheimer H. (2001). Reduced expression of connexin-43 and functional gap junction coupling in human gliomas. Glia.

[35] Eng L.F. (1985). Glial fibrillary acidic protein (GFAP): The major protein of glial intermediate filaments in differentiated astrocytes. J. Neuroimmunol..

[36] Xu D., Wang Q., Gruber A., Björkholm M., Chen Z., Zaid A., Selivanova G., Peterson C., Wiman K.G., Pisa P. (2000). Downregulation of telomerase reverse transcriptase mRNA expression by wild type p53 in human tumor cells. Oncogene.

[37] Kurki P., Vanderlaan M., Dolbeare F., Gray J., Tan E.M. (1986). Expression of proliferating cell nuclear antigen (PCNA)/cyclin during the cell cycle. Exp. Cell Res..

[38] Johansson C.B., Momma S., Clarke D.L., Risling M., Lendahl U., Frisén J. (1999). Identification of a Neural Stem Cell in the Adult Mammalian Central Nervous System. Cell.

[39] Sakakibara S.-I., Imai T., Hamaguchi K., Okabe M., Aruga J., Nakajima K., Yasutomi D., Nagata T., Kurihara Y., Uesugi S. (1996). Mouse-Musashi-1, a Neural RNA-Binding Protein Highly Enriched in the Mammalian CNS Stem Cell. Dev. Biol..

[40] Singh S.K., Hawkins C., Clarke I.D., Squire J.A., Bayani J., Hide T., Henkelman R.M., Cusimano M.D., Dirks P.B. (2004). Identification of human brain tumour initiating cells. Nat. Cell Biol..

[41] Tanabe K., Matsushima-Nishiwaki R., Yamaguchi S., Iida H., Dohi S., Kozawa O. (2010). Mechanisms of tumor necrosis factor-α-induced interleukin-6 synthesis in glioma cells. J. Neuroinflamm..

[42] Gupta P.B., Chaffer C.L., Weinberg R.A. (2009). Cancer stem cells: Mirage or reality?. Nat. Med..

[43] Shackleton M., Quintana E., Fearon E.R., Morrison S.J. (2009). Heterogeneity in Cancer: Cancer Stem Cells versus Clonal Evolution. Cell.

[44] Aboody K.S., Brown A.B., Rainov N.G., Bower K.A., Liu S., Yang W., Small J.E., Herrlinger U., Ourednik V., Black P.M. (2000). Neural stem cells display extensive tropism for pathology in adult brain: Evidence from intracranial gliomas. Proc. Natl. Acad. Sci. USA.

[45] Park J., Kim C.G., Shim J.K., Kim J.H., Lee H., Lee J.E., Kim M.H., Haam K., Jung I., Park S.H. (2018). Effect of combined anti-PD-1 and temozolomide therapy in glioblastoma. Oncoimmunology.

[46] Cammarata F.P., Torrisi F., Forte G.I., Minafra L., Bravatà V., Pisciotta P., Savoca G., Calvaruso M., Petringa G., Cirrone G.A.P. (2019). Proton Therapy and Src Family Kinase Inhibitor Combined Treatments on U87 Human Glioblastoma Multiforme Cell Line. Int. J. Mol. Sci..

[47] Islam O., Gong X., Rose-John S., Heese K. (2009). Interleukin-6 and Neural Stem Cells: More Than Gliogenesis. Mol. Biol. Cell.

[48] Taga T., Nakashima K. (2002). Mechanisms Underlying Cytokine-Mediated Cell-Fate Regulation in the Nervous System. Mol. Neurobiol..

[49] Barkho B.Z., Song H., Aimone J.B., Smrt R.D., Kuwabara T., Nakashima K., Gage F.H., Zhao X. (2006). Identification of Astrocyte-expressed Factors That Modulate Neural Stem/Progenitor Cell Differentiation. Stem Cells Dev..

[50] Gallagher D., Norman A.A., Woodard C.L., Yang G., Gauthier-Fisher A., Fujitani M., Vessey J.P., Cancino G.I., Sachewsky N., Woltjen K. (2013). Transient Maternal IL-6 Mediates Long-Lasting Changes in Neural Stem Cell Pools by Deregulating an Endogenous Self-Renewal Pathway. Cell Stem Cell.

[51] Hossain A., Gumin J., Gao F., Figueroa J., Shinojima N., Takezaki T., Priebe W., Villarreal D., Kang S.G., Joyce C. (2015). Mesenchymal stem cells isolated from human gliomas increase proliferation and maintain stemness of glioma stem cells through the IL-6/gp130/STAT3 pathway. Stem Cells.

[52] Plaks V., Kong N., Werb Z. (2015). The Cancer Stem Cell Niche: How Essential Is the Niche in Regulating Stemness of Tumor Cells?. Cell Stem Cell.

[53] Benveniste E.N., Sparacio S.M., Norris J.G., Grennett H.E., Fuller G.M. (1990). Induction and regulation of interleukin-6 gene expression in rat astrocytes. J. Neuroimmunol..

[54] Benveniste E.N., Kwon J., Chung W.J., Sampson J., Pandya K., Tang L.P. (1994). Differential modulation of astrocyte cytokine gene expression by TGF-beta. J. Immunol..

[55] Lee J., Kotliarova S., Kotliarov Y., Li A., Su Q., Donin N.M., Pastorino S., Purow B.W., Christopher N., Zhang W. (2006). Tumor stem cells derived from glioblastomas cultured in bFGF and EGF more closely mirror the phenotype and genotype of primary tumors than do serum-cultured cell lines. Cancer Cell.

[56] Li A., Walling J., Kotliarov Y., Center A., Steed M.E., Ahn S.J., Rosenblum M., Mikkelsen T., Zenklusen J.C., Fine H.A. (2008). Genomic Changes and Gene Expression Profiles Reveal That Established Glioma Cell Lines Are Poorly Representative of Primary Human Gliomas. Mol. Cancer Res..

[57] Tirino V., Desiderio V., d’Aquino R., De Francesco F., Pirozzi G., Graziano A., Galderisi U., Cavaliere C., De Rosa A., Papaccio G. (2008). Detection and characterization of CD133+ cancer stem cells in human solid tumours. PLoS ONE.

[58] Beier D., Hau P., Proescholdt M.A., Lohmeier A., Wischhusen J., Oefner P.J., Aigner L., Brawanski A., Bogdahn U., Beier C.P. (2007). CD133+ and CD133- Glioblastoma-Derived Cancer Stem Cells Show Differential Growth Characteristics and Molecular Profiles. Cancer Res..

[59] Bonnet D., Dick J.E. (1997). Human acute myeloid leukemia is organized as a hierarchy that originates from a primitive hematopoietic cell. Nat. Med..

[60] Kelly P.N., Dakic A., Adams J.M., Nutt S.L., Strasser A. (2007). Tumor Growth Need Not Be Driven by Rare Cancer Stem Cells. Science.

[61] Quintana E., Shackleton M., Sabel M.S., Fullen D.R., Johnson T.M., Morrison S.J. (2008). Efficient tumour formation by single human melanoma cells. Nat. Cell Biol..

[62] Kreso A., Dick J.E. (2014). Evolution of the Cancer Stem Cell Model. Cell Stem Cell.

