# Supplementary figures and images for: Differentiation of Tumorigenic C6 Glioma Cells Induced by Enhanced IL-6 Signaling
Source: Medicina (Kaunas). 2020 Nov 19;56(11):625. doi: 10.3390/medicina56110625 (PMC7699282; doi:10.3390/medicina56110625)

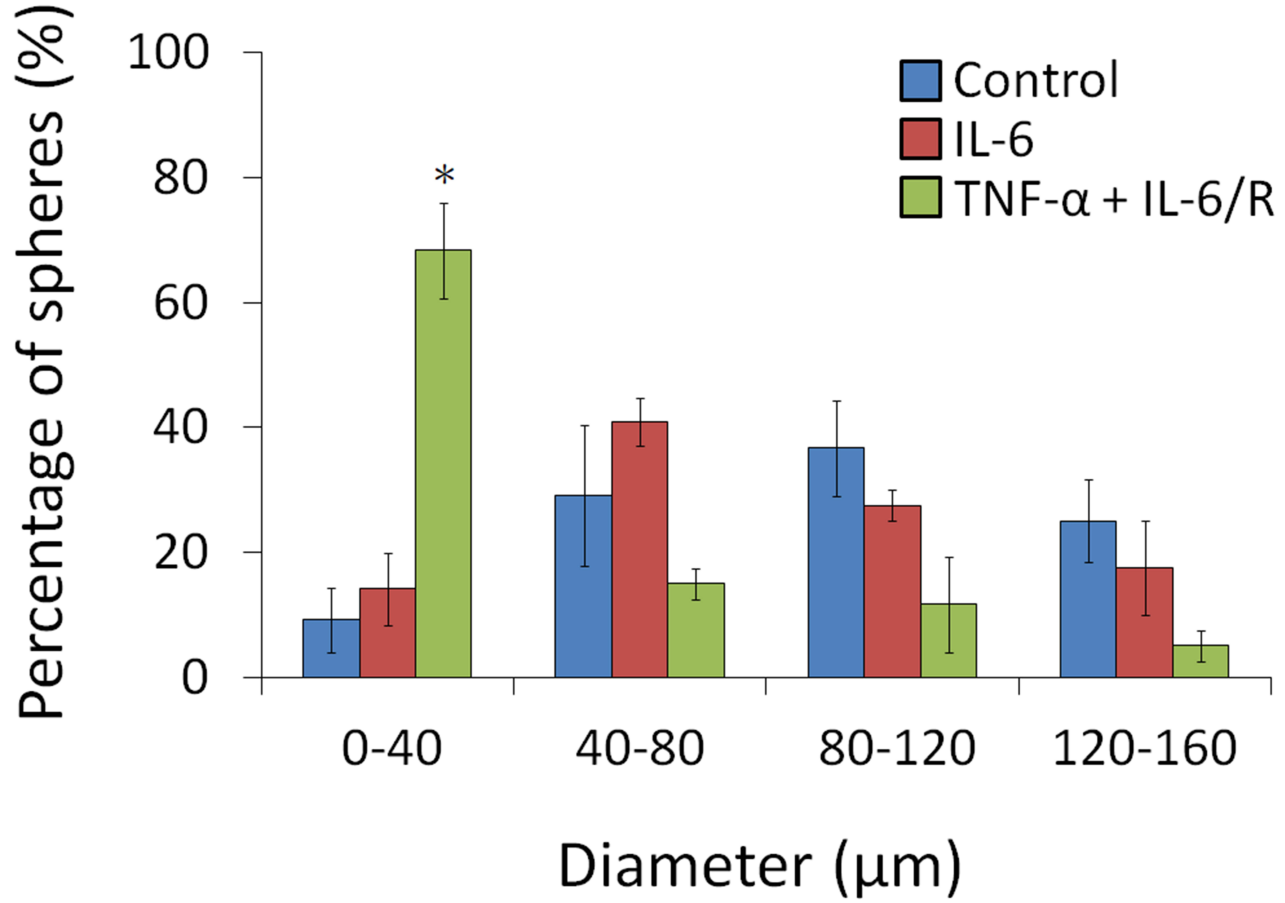

Supplement: Supplementary file 1 [file medicina-56-00625-s001.pdf]
